# Evaluating the Role of Glycemic Control in Modulating Pulmonary Function Among Smokers With Diabetes Mellitus: A Systematic Review

**DOI:** 10.7759/cureus.56895

**Published:** 2024-03-25

**Authors:** Tanuj Mathur, Bipin Kumar, Mamta Dubey, Krishna Keerthi Annepu, Yoshita Rao Annepu, Shivakumar G C

**Affiliations:** 1 Department of Physiology, Jawaharlal Nehru Medical College, Aligarh Muslim University, Aligarh, IND; 2 Department of Physiology, Motilal Nehru Medical College, Prayagraj, Prayagraj, IND; 3 Medicine, Rangaraya Medical College, Kakinada, IND; 4 Department of Oral Medicine and Radiology, People’s College of Dental Science and Research Centre, Bhopal, IND

**Keywords:** hba1c, glycemic control, smoking, pulmonary function, diabetes mellitus

## Abstract

Background: Diabetes mellitus (DM) impacts multiple body systems, including lung function, and this impact can be further complicated by smoking. The connection between blood sugar control and lung health in individuals with diabetes who smoke has been extensively studied, but findings have been varied. This systematic review sought to compile and assess the research on how blood sugar control influences lung function in smokers with diabetes.

Methods: We searched several databases, including PubMed, EMBASE, Cochrane Library, Web of Science, Scopus, CINAHL, PsycINFO, and Google Scholar, in line with Preferred Reporting Items for Systematic Reviews and Meta-Analyses (PRISMA) guidelines. We included studies that looked at lung function tests in smokers with diabetes and examined the relationship with blood sugar control, as indicated by hemoglobin A1c (HbA1c) levels. We conducted thorough quality assessments, data extraction, and analysis.

Results: We identified five relevant studies. The data from these studies indicated a clear trend: smokers with diabetes who had higher HbA1c levels typically showed worse lung function than those with better blood sugar control. Decreases in forced expiratory volume in one second (FEV1) and forced vital capacity (FVC) were the most frequently observed issues. Some studies also pointed to a complex relationship between HbA1c levels and lung function, particularly when HbA1c was below 7.0%.

Conclusion: Our review indicates that smokers with DM who have poor blood sugar control tend to have worse lung function. These findings highlight the importance of managing blood sugar to help maintain lung health in these individuals. Further long-term research is needed to clarify the exact relationship and whether improving blood sugar control can reverse lung problems.

## Introduction and background

Diabetes mellitus (DM) is a chronic metabolic disorder characterized by hyperglycemia resulting from defects in insulin secretion, insulin action, or both [1]. The global prevalence of diabetes is escalating, and it is projected to affect 629 million individuals by 2045 [1-2]. The complications of DM are multifarious and can affect multiple organ systems, including the cardiovascular, renal, and nervous systems [3]. An emerging body of evidence suggests that pulmonary function is also compromised in individuals with DM, manifesting in reduced lung volumes and impaired gas exchange capabilities [4]. The nexus between DM and pulmonary dysfunction is hypothesized to be influenced by chronic hyperglycemia-induced microvascular damage, leading to alterations in the lung parenchyma and airway remodeling. Moreover, the diabetic lung is often characterized by a reduced capacity to counteract inflammatory processes, which can exacerbate structural and functional pulmonary changes [5]. Smoking is a well-known risk factor for both the inception and progression of pulmonary diseases, such as chronic obstructive pulmonary disease (COPD) and lung cancer, and it further aggravates the inflammatory milieu of the diabetic lung [6]. The interplay between DM, smoking, and lung function is intricate and remains an area of active research. Smokers with diabetes represent a unique subset of individuals who are especially vulnerable to pulmonary complications [7].

Hyperglycemia has also been implicated in altering the composition of airway surface liquid (ASL), specifically by increasing glucose concentration within this fluid [8]. This alteration has been associated with an enhanced propensity for bacterial proliferation [9], which in turn may lead to an escalation in pulmonary complications [10-11]. Such complications can precipitate acute healthcare utilization, including emergency department visits and hospital admissions, with subsequent hospital courses often associated with suboptimal outcomes. Additionally, hyperglycemia may impair pulmonary innate immunity. This impairment has been linked to a decrease in surfactant protein D, a critical component of the lung's defense system, potentially leading to an increased incidence of respiratory infections and a decline in the diffusing capacity of the lungs. Moreover, smoking has been identified as an independent risk factor for the development of type 2 diabetes [11], and cessation of smoking has been correlated with a reduced risk of developing metabolic syndrome. Beyond the direct effects of tobacco use, other pulmonary risk factors, such as diminished lung function, limited exercise capacity, heightened experiences of dyspnea, and a greater susceptibility to respiratory infections, have been associated with an increased risk of developing diabetes. Glycemic control, typically quantified by the level of hemoglobin A1c (HbA1c), is a cornerstone in the management of DM and has been postulated to affect pulmonary function. While there is consensus on the benefits of glycemic control in mitigating the microvascular complications of diabetes, its role in modulating lung function, particularly among smokers with DM, is less clear [9-11]. This systematic review aims to critically appraise and synthesize existing literature on the role of glycemic control in modulating pulmonary function among smokers with DM and investigate whether the two are interlinked and, if so, to what degree. By elucidating the relationship between glycemic control and pulmonary outcomes, this review seeks to inform clinical practice and guide future research in the management of lung health in this high-risk population.

## Review

Eligibility criteria

The Preferred Reporting Items for Systematic Reviews and Meta-Analyses (PRISMA) reporting guidelines [12] were adhered to in this systematic review. The PECO (Population, Exposure, Comparator, Outcome) protocol for the systematic review was defined as follows.

Population (P): Adult smokers with a clinical diagnosis of DM were the primary population of interest. This included individuals with both types of DM who were current smokers at the time of the studies or had a history of smoking, Exposure (E): The primary exposure of interest was the level of glycemic control, which could be represented by various measures such as HbA1c levels, fasting blood glucose, or postprandial glucose levels, Comparator (C): Although a comparator was not mandatory considering the aims and scope of the review, studies that included non-smoking diabetics, non-diabetic smokers, or smokers with diabetes with different levels of glycemic control as comparators were welcomed and included wherever present. This approach allowed for a broader understanding of the relative effects of glycemic control on pulmonary function in the context of smoking, Outcome (O): The primary outcomes of interest were measures of pulmonary function, which included but were not limited to forced expiratory volume in one second (FEV1), forced vital capacity (FVC), peak expiratory flow (PEF), and diffusing capacity of the lungs for carbon monoxide (DLCO). Secondary outcomes might have included the incidence of respiratory symptoms, pulmonary exacerbations, or quality-of-life measures related to respiratory health.

Table 1 represents the eligibility criteria devised for this review.

**Table 1:** Inclusion and exclusion criteria devised for this review FEV1: forced expiratory volume in one second; FVC: forced vital capacity; PEF: peak expiratory flow; DLCO: diffusing capacity of the lungs for carbon monoxide

Criteria	Inclusion	Exclusion
Population	Adults diagnosed with type 1 or type 2 diabetes mellitus who were current smokers or had a history of smoking.	Non-adults (e.g., children, adolescents), individuals without a clinical diagnosis of diabetes mellitus, non-smokers, and former smokers with no recent history of smoking.
Exposure	Studies that specifically measured glycemic control using HbA1c levels, fasting blood glucose, postprandial glucose levels, or any other clinically recognized methods.	Studies not focusing on the assessment of glycemic control or using non-standardized/validated measures of glycemic control.
Comparator	Studies with or without a comparator group, including non-smoking diabetics, non-diabetic smokers, or smokers with different levels of glycemic control.	Studies that did not provide clear comparative data when a comparator group was utilized.
Outcomes	Studies that included measures of pulmonary function such as FEV1, FVC, PEF, DLCO, or reported respiratory symptoms, pulmonary exacerbations, or respiratory-related quality of life.	Studies that did not include pulmonary function measures or related clinical outcomes.
Study Design	Randomized controlled trials (RCTs), cohort studies, case-control studies, and cross-sectional studies.	Reviews, editorials, case reports, animal studies, and studies with insufficient methodological details.
Language	No limitation placed	

Database search protocol

The database search protocol for this review was developed using Boolean operators and Medical Subject Headings (MeSH) keywords to identify relevant studies. The search strategy was tailored to each database's unique syntax and search capabilities to ensure thorough literature retrieval. The databases searched included PubMed, EMBASE, Cochrane Library, Web of Science, Scopus, CINAHL, PsycINFO, and Google Scholar. The search strings combined terms related to DM, smoking, glycemic control, and pulmonary function. Boolean operators ("AND" and "OR") were employed to combine keywords and MeSH terms where appropriate, as represented in Table 2. The search was designed to be both sensitive and specific, capturing the broadest possible range of relevant studies while filtering out unrelated literature.

**Table 2:** Search strings utilized across the databases FEV1: forced expiratory volume in one second; FVC: forced vital capacity

Database	Search String
PubMed	("Diabetes Mellitus"[MeSH Terms] OR diabetes) AND ("Smoking"[MeSH Terms] OR smoker) AND ("Glycemic Control"[MeSH Terms] OR "Blood Glucose"[MeSH Terms] OR HbA1c) AND ("Pulmonary Function Tests"[MeSH Terms] OR "Respiratory Function Tests"[MeSH Terms] OR FEV1 OR FVC)
EMBASE	(“diabetes mellitus”/exp OR diabetes) AND (“smoking”/exp OR smoker) AND (“glycemic control”/exp OR “blood glucose”/exp OR HbA1c) AND (“pulmonary function test”/exp OR “respiratory function test”/exp OR FEV1 OR FVC)
Cochrane Library	(MeSH descriptor: [Diabetes Mellitus] OR diabetes) AND (MeSH descriptor: [Smoking] OR smoker) AND (glycemic control OR blood glucose OR HbA1c) AND (MeSH descriptor: [Pulmonary Function Tests] OR FEV1 OR FVC)
Web of Science	(TI=(diabetes) OR TS=(Diabetes Mellitus)) AND (TI=(smoking) OR TS=(Smoker)) AND (TI=(glycemic control) OR TS=(Blood Glucose) OR TS=(HbA1c)) AND (TI=(pulmonary function) OR TS=(FEV1) OR TS=(FVC))
Scopus	(TITLE-ABS-KEY (diabetes mellitus) OR TITLE-ABS-KEY (diabetes)) AND (TITLE-ABS-KEY (smoking) OR TITLE-ABS-KEY (smoker)) AND (TITLE-ABS-KEY (glycemic control) OR TITLE-ABS-KEY (blood glucose) OR TITLE-ABS-KEY (HbA1c)) AND (TITLE-ABS-KEY (pulmonary function tests) OR TITLE-ABS-KEY (FEV1) OR TITLE-ABS-KEY (FVC))
CINAHL	(MH "Diabetes Mellitus" OR diabetes) AND (MH "Smoking" OR smoker) AND (MH "Glycemic Control" OR "Blood Glucose" OR HbA1c) AND (MH "Pulmonary Function Tests" OR FEV1 OR FVC)
PsycINFO	(DE "Diabetes Mellitus" OR diabetes) AND (DE "Tobacco Use" OR smoker) AND (glycemic control OR blood glucose OR HbA1c) AND (DE "Respiratory Function Tests" OR FEV1 OR FVC)
Google Scholar	allintitle: diabetes mellitus smoking glycemic control pulmonary function FEV1 FVC

Data extraction protocol

A standardized data extraction form was developed and pilot-tested on a subset of included studies to ensure its comprehensiveness and functionality. The form was designed to capture all pertinent study characteristics and outcome measures, like bibliographic details (author names, year of publication, journal), study design and methodology, participant demographics (age, gender, smoking history, type and duration of diabetes), intervention and comparator details (if applicable), outcome measures (types of pulmonary function tests used, results, and time points), statistical analysis methods, and confounders and effect modifiers considered. Two independent reviewers conducted the data extraction process to minimize bias and errors. They were thoroughly trained on the use of the form and the specific data extraction procedures. The reviewers extracted data independently and subsequently compared their findings. Disagreements were resolved through discussion, and if consensus could not be reached, a third reviewer was consulted.

Bias assessment tools

In evaluating the potential for bias within this review, we applied two recognized evaluation instruments. The AXIS tool [13] was used to appraise cross-sectional study designs, while the ROBINS-I framework [14] was employed for the examination of non-randomized studies. Firstly, the AXIS tool [13] was utilized to assess cross-sectional study designs. The AXIS tool is recognized for its capacity to systematically evaluate the quality of cross-sectional studies, focusing on critical aspects such as methodology, sample selection, and data analysis. It provides a structured approach to identifying potential biases and limitations inherent in cross-sectional research, aiding reviewers in their appraisal process. Second, the review utilized the ROBINS-I framework [14], referenced by citation [14], to scrutinize non-randomized studies. This tool is a widely acknowledged tool designed specifically for assessing the risk of bias in non-randomized studies of interventions. It comprehensively evaluates various domains, including bias due to confounding, selection bias, and information bias, among others.

Certainty bias assessment

Following the initial bias evaluations, we adopted the GRADE (Grading of Recommendations, Assessment, Development, and Evaluation) methodology [15] to determine the reliability of the evidence gathered from the included studies. The GRADE approach was used after completing the individual study bias assessments. It assesses the collective quality of evidence from the studies for specific outcomes by considering various elements: the extent of bias, consistency of findings, directness of evidence, accuracy of the effect estimates, and the likelihood of publication bias. These aspects help in assigning the evidence a grade that ranges from high to very low in terms of reliability.

Study selection process

**Figure 1:**
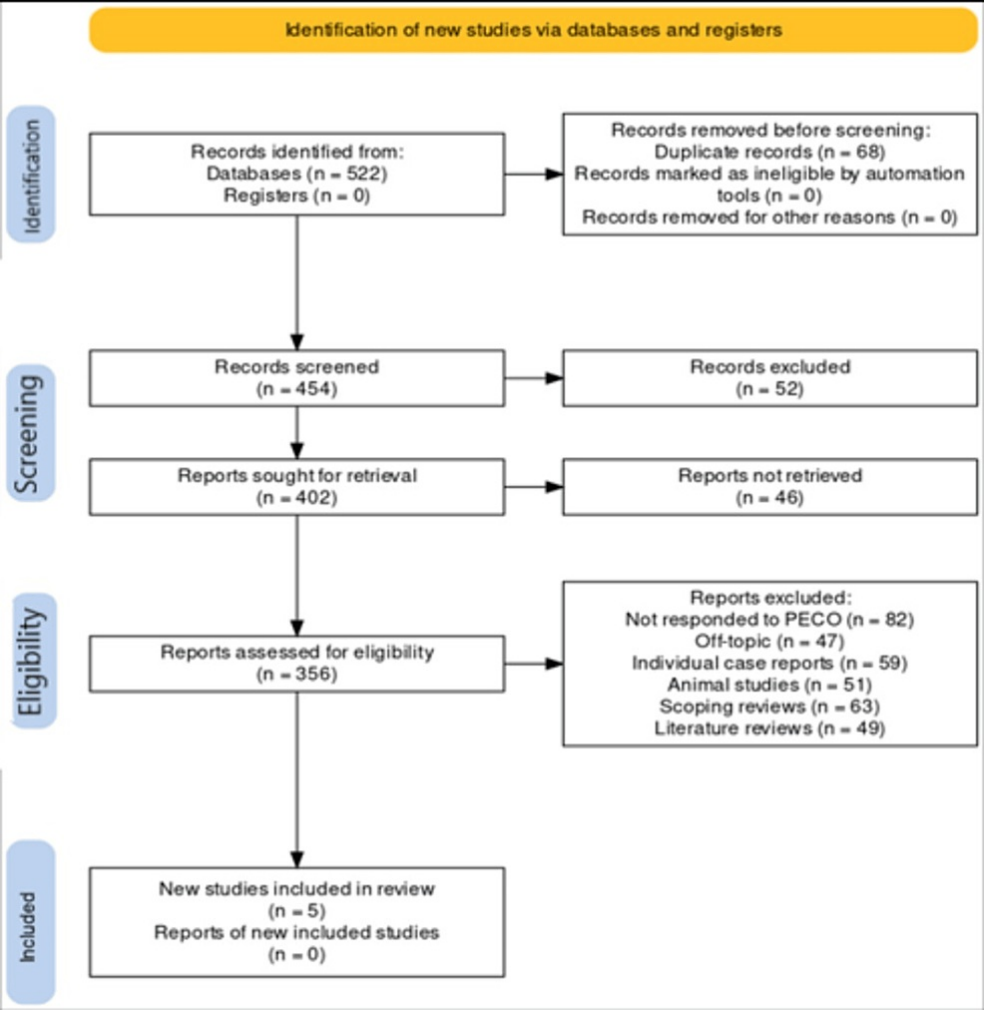
Different stages of the article identification and screening for this review The flowchart has been prepared by the authors of this article.

The process of study selection is depicted in Figure 1, beginning with the initial retrieval of 522 records from the selected databases. There were no records obtained from the registry. Subsequent to the removal of 68 duplicates, the remaining 454 records underwent screening. Access issues led to the exclusion of 52 records, leaving 402 records available for further consideration. From these, 46 records could not be secured for detailed assessment. The remaining 356 records were subject to an in-depth eligibility review. During this phase, 47 records were excluded for being irrelevant to the research question, while 82 records did not conform to the predefined PICO (population, interventions, comparators, and outcomes) criteria. This review phase also removed 49 narrative reviews, 51 animal studies, and 59 scoping reviews due to their incompatibility with the inclusion criteria. After applying these criteria, five papers [16-20] were identified as suitable for inclusion in the systematic review.

Assessed bias across different domains

**Figure 2:**
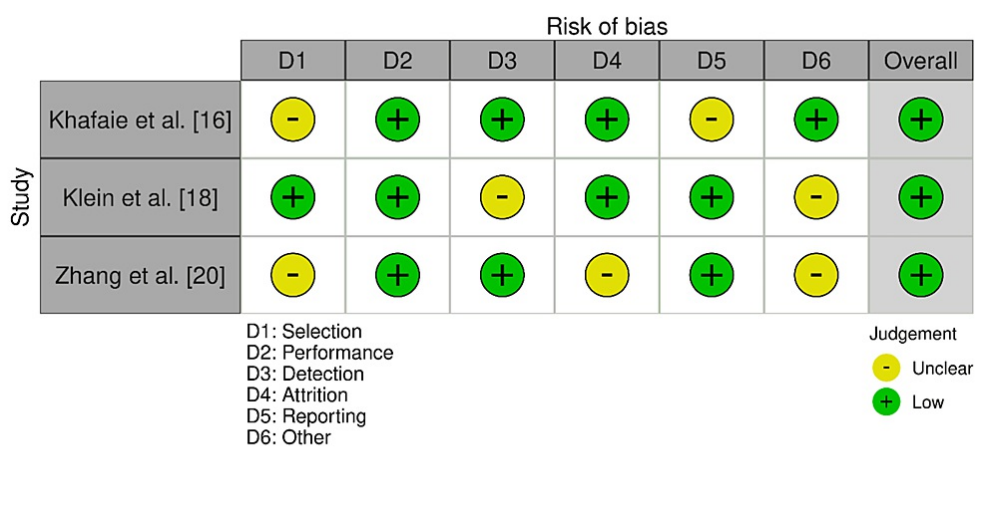
Bias assessed across the cross-sectional papers included in the review The image has been prepared by the authors of this article.

For the studies evaluated with the AXIS tool (Figure 2), Khafaie et al. [16] presented a moderate risk of bias in selection and reporting but a low risk in performance, detection, attrition, and other biases. Klein et al. [18] had a low risk of bias across selection, performance, detection, and attrition, with moderate risks in detection and reporting. Zhang et al. [20] showed a moderate risk in selection and detection, with a low risk in performance, attrition, and other biases, but a moderate risk in reporting. Each of these studies was ultimately adjudged to have an overall low risk of bias.

**Figure 3:**
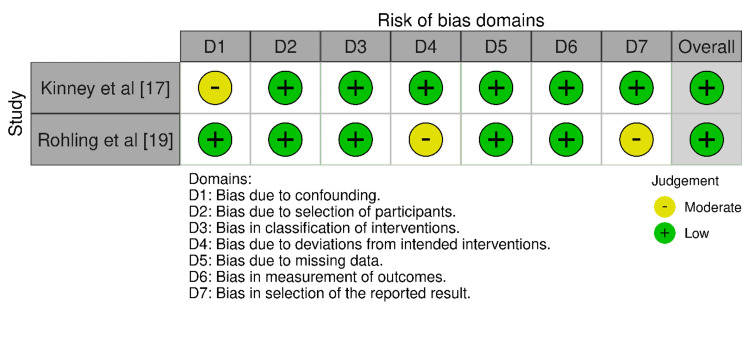
Bias assessed across the case-control and cohort-based studies included in the review The image has been prepared by the authors of this article.

Using the ROBINS-I tool (Figure 3), Kinney et al. [17] found a moderate risk of bias in domain 1 (D1), which pertains to confounding variables, but low risk across the other domains (D2-D7). The overall bias was considered low. Röhling et al. [19] had a low risk of bias in domains D1-D3 and D5-D6, with moderate risks in domains D4, related to missing data, and D7, concerning the selection of reported results. This study was also given an overall low risk of bias rating.

Demographic variables assessed

Table 3 outlines the research articles scrutinized in this systematic review [16-20]. The article by Khafaie et al. [16] executed a cross-sectional analysis on 865 subjects, splitting them into 400 individuals with T2DM and 465 without. The study concentrated on chronic respiratory complaints and involved spirometry to assess lung capacity. This method facilitated concurrent respiratory evaluation in both diabetic and non-diabetic groups to discern disparities or associations.

The work by Kinney et al. [17] was based on a longitudinal cohort model, with an extensive participant count of 7,080 from the COPDGene project, tracking both current and former smokers over 4.2 years. This long-term study model was crucial for monitoring respiratory health evolution and detecting smoking's effects on lung capacity in the context of developing diabetes. Klein et al. [18] conducted a cross-sectional analysis of 4,164 adults aged 18 to 97, sourced from a patient pool at a medical center, within the period from January 2000 to May 2009. The selection process omitted individuals with known lung function impairments unrelated to diabetes, thus sharpening the focus on the diabetes-lung function interplay. The study by Röhling et al. [19] embraced a case-control setup with 60 subjects, comparing 34 individuals newly diagnosed with type 2 diabetes against 26 overweight but non-diabetic controls. The case-control format was apt for examining the immediate effects of type 2 diabetes on respiratory function and differentiating the respiratory health between people with recent diabetes diagnoses and overweight non-diabetics. Zhang et al. [20] undertook a cross-sectional study with a broad participant base of 8,584 adults from NHANES spanning 2007-2012. This study's design capitalized on a demographically diverse sample from the USA to explore connections among diabetes, respiratory function, and other metabolic indicators.

**Table 3:** Studies included in the review and their associated inferences PRISm: patient-reported impact of sickness; FEV1: forced expiratory volume in one second; FVC: forced vital capacity; PEF: peak expiratory flow; DLCO: diffusing capacity of the lungs for carbon monoxide; CRSs: chronic respiratory symptoms; COPD: chronic obstructive pulmonary disease; PFT: pulmonary function test

Study ID	Study Design	Sample Size	Participant Characteristics	Key Variables and Measures	Analysis Method	Significant Findings	Overall Inference Drawn
Khafaie et al. 2017 [16]	Cross-sectional	865	400 type 2 diabetic and 465 healthy subjects investigated for CRSs and underwent spirometry.	CRSs, FVC, FEV1, FEV1%, FVC%, PM_10_ exposure estimated using AERMOD. Logistic and linear regression models to explore association between PM_10_ and CRSs, FEV1%, FVC% with adjustments for confounders	Logistic regression for CRSs; linear regression for FEV1% and FVC%	Diabetic subjects had significantly higher prevalence of wheezing, allergy symptoms, chest tightness, and physician-diagnosed asthma and COPD. No significant difference in the percent predicted value of PFT between diabetic and non-diabetic subjects. 1 SD increase in PM_10_ concentration associated with a 1.50-fold (95% CI, 1.12–2.01) greater risk of dyspnea and a 3.71% (95% CI, 0.48–4.99) decrease in FVC%. Associations were stronger in overweight, smokers, and older individuals. Independent contribution of air pollution to reduced lung function regardless of diabetes status.	Air pollution, particularly PM_10_, contributes to reduced lung function and is a significant risk factor for CRSs, with a more pronounced effect in individuals with diabetes, especially if they are overweight, smokers, or older.
Kinney et al. 2016 [17]	Longitudinal cohort	7080	Participants from the COPDGene study, current and former smokers, 4.2 years follow-up	Incident diabetes, FEV1, FVC, FEV1/FVC, respiratory exacerbations, 6MWD, corticosteroid use, chronic bronchitis, dyspnea, PRISm	Cox proportional hazards modeling	Current smoking, BMI, high blood pressure, high cholesterol, and pulmonary markers are associated with increased risk of incident diabetes.	Pulmonary function and exacerbations are associated with the development of diabetes in smokers, indicating a potential area for intervention to prevent diabetes.
Klein et al. 2012 [18]	Cross-sectional	4164	Adults aged 18–97 from a medical center (1 January 2000 to 1 May 2009), excluding those with diseases causing abnormal lung function.	Diabetes mellitus, FVC, FEV1, DLCO, age, sex, race, height, smoking status, BMI, heart failure	Multiple linear regression	Unadjusted and adjusted values showed significantly lower FVC, FEV1, and DLCO in patients with diabetes. These differences remained after adjusting for covariates. Stratification by race showed only Caucasians with diabetes had significant decreases in all lung function measures. Adjusted values also showed significant differences when stratified by heart failure and age groups, with poorer pulmonary function in patients with diabetes across all stratifications.	The presence of diabetes is associated with decreased pulmonary function in smokers, and this relationship is influenced by race, heart failure status, and age.
Röhling et al. 2018 [19]	Case-control	60	Type 2 diabetes patients with disease duration <1 year (n=34) compared with overweight controls (n=26).	FVC%, FEV1%, FEV1/FVC ratio, SNP genotyping, VO2max, HbA1c levels, hs-CRP. Multivariable linear regressions adjusted for confounders such as age, sex, BMI, height, smoking status	Multivariable linear regressions	Patients with type 2 diabetes had lower FEV1%, FEV1/FVC, and VO2max. Positive correlation between FEV1% and VO2max, and a negative correlation between FEV1/FVC and HbA1c in diabetic patients. Group differences in FEV1/FVC could be explained by HbA1c. No significant difference in FVC% between the groups.	HbA1c levels in patients with type 2 diabetes appear to be a confounding factor associated with reduced lung function as evidenced by the FEV1/FVC ratio. Glycemic control may play a role in pulmonary function among patients with recent-onset diabetes.
Zhang et al. 2020 [20]	Cross-sectional	8584	Adults from the National Health and Nutrition Examination Survey (2007-2012), U.S. population	Diabetes mellitus, FEV1, FVC, HbA1c, fasting plasma glucose, 2 h-plasma glucose, insulin resistance, CRP, obesity	Multiple linear regression, restricted cubic spline analysis, partial mediation analysis	Diabetes associated with reduced FEV1 and FVC; L-shaped associations between HbA1c and FEV1/FVC for those with HbA1c	Strict glycemic control might improve pulmonary function in diabetic smokers. Diabetes duration did not affect pulmonary function.

Pulmonary parameters and variables measured

Khafaie et al. [16] quantified the impact of PM_10_ on respiratory symptoms and lung capacity through regression analyses, taking into account potential confounders. This meticulous approach allowed for a nuanced understanding of the respiratory risks associated with air pollution, particularly among diabetic populations. Kinney et al. [17] delved into the predictive capability of lung health indicators for diabetes onset, utilizing survival analysis models. By employing these models, they provided insights into how respiratory function may precede and predict the development of diabetes, highlighting the importance of considering longitudinal data in understanding disease progression. Klein et al. [18] employed regression techniques to evaluate the effects of diabetes on lung capacity, considering various demographic and health factors. By adjusting for these factors, they conducted a thorough evaluation of diabetes' influence across a diverse adult demographic, enhancing the robustness of their findings.

Röhling et al. [19] conducted a case-control study analyzing genetic and physiological markers alongside lung function measurements, adjusting for various covariates. This comprehensive approach allowed them to identify a significant link between glucose control and lung health in early-stage type 2 diabetes, highlighting the importance of considering genetic and physiological factors in understanding the relationship between diabetes and respiratory health. Zhang et al. [20] utilized advanced statistical methods to unravel the complex relationships between diabetes, metabolic control, and respiratory capacity on a large population scale. By employing these sophisticated techniques, they enhanced our understanding of the intricate interactions between these variables, providing valuable insights into the mechanisms underlying the association between diabetes and pulmonary function. In overall terms, while differences in measurement techniques existed across studies, the standardized analysis and adjustment for confounding variables ensured a rigorous evaluation of the relationship between glycemic control and pulmonary function among smokers with DM, facilitating a comprehensive understanding of these complex interactions.

Findings observed

Khafaie et al. [16] noted an increased prevalence of respiratory symptoms among individuals with diabetes, yet they did not find significant differences in lung function measures compared to their non-diabetic counterparts. However, they identified a correlation between elevated ambient PM_10_ levels and respiratory discomfort, particularly among subpopulations with additional risk factors, which might have confounded the observed associations. Similarly, Kinney et al. [17] identified lifestyle and health markers such as smoking status and cardiovascular risk factors as contributors to the development of diabetes, indicating a complex interplay between these factors and metabolic disease progression. This complexity could introduce confounding variables that influence the relationship between glycemic control and pulmonary function. Klein et al. [18] reported lower lung function in diabetic patients, even after adjusting for various factors. The differential impact of diabetes on lung capacity across demographic groups underscores the importance of considering potential confounding variables related to age, sex, and comorbidities. Röhling et al. [19] observed reduced lung function and exercise capacity in diabetic subjects, highlighting the influence of metabolic control on respiratory outcomes. However, the specificity of these changes to diabetes requires careful consideration of potential confounders such as medication usage and lifestyle factors. Zhang et al. [20] revealed a nonlinear relationship between glycemic control and lung capacity, suggesting a potential glycemic threshold beyond which lung function may be impacted. Yet, factors such as medication adherence and comorbid conditions could confound this relationship, necessitating careful interpretation of the findings.

GRADE assessment observations

The GRADE certainty assessment of the studies under review indicates that the evidence is of low to moderate certainty (Table 4). This assessment is based on the study designs, with three cross-sectional studies, one longitudinal cohort study, and one case-control study. The observed common finding across these studies is that air pollution, particularly PM10, and the presence of diabetes are associated with decreased lung function. The risk of bias was considered low to moderate for the cross-sectional and case-control studies due to the inherent limitations of these observational designs, which do not allow for establishing causality. There is a low level of inconsistency, as most studies reported similar findings, supporting the association between diabetes and reduced lung function. Indirectness was assessed as low because the studies directly measured the outcomes of interest (lung function) in the populations of interest (individuals with diabetes and smokers). Imprecision was rated as low to moderate due to the limited number of studies and the potential variability in their results. No other domains were deemed to significantly impact the certainty of the evidence.

**Table 4:** GRADE assessment observations for certainty bias GRADE: Grading of Recommendations, Assessment, Development, and Evaluation

Study Design	Number Of Studies	Observed Common Finding	Risk of Bias	Inconsistency	Indirectness	Imprecision	Others	Certainty
Cross-sectional	3	Air pollution contributes to reduced lung function; diabetes presence is associated with decreased pulmonary function in smokers.	Low to moderate	Low	Low	Low to moderate	None	Low to moderate
Longitudinal cohort	1	Pulmonary function and exacerbations are associated with the development of diabetes in smokers.	Low	Not applicable	Low	Low	None	Moderate
Case-control	1	HbA1c levels are associated with reduced lung function in type 2 diabetes.	Low to moderate	Not applicable	Low	Low to moderate	None	Low to moderate

Upon the comparative analysis of the collective findings from the included articles [16-20], distinct patterns and correlations emerged, as well as certain dissimilarities. The study by Khafaie et al. [16] underscored the external environmental factor of air pollution, particularly PM10, as a significant contributor to reduced lung function, with diabetes exacerbating this effect. The findings were somewhat aligned with those presented by Kinney et al. [17], who also emphasized the role of external factors-in this case, smoking-on the development of diabetes and its subsequent impact on pulmonary function. Both studies [16, 17] highlighted the interaction between environmental exposures and metabolic disease, suggesting that diabetes may sensitize individuals to the deleterious effects of pollutants and smoking on lung function. However, the focus of Khafaie et al. [16] on air pollution as a universal risk factor differed from the patient-specific risk factor of smoking in Kinney et al. [17]. Klein et al. [18] reported that diabetes was associated with declines in pulmonary function, identifying particular vulnerability based on race, heart failure status, and age. This study's outcomes were congruent to some extent with the results from Khafaie et al. [16] and Kinney et al. [17] in that all three studies [16, 17, 18] acknowledged the additive or synergistic effect of diabetes and other factors on lung function. However, Klein et al. [18] diverged in highlighting intrinsic factors such as race and heart failure as modifiers of the diabetes-lung function relationship, whereas the other studies focused more on extrinsic factors. Röhling et al. [19] identified HbA1c as a confounding factor linked to reduced lung function, specifically the FEV1/FVC ratio, in type 2 diabetes. This internal metabolic control factor is a point of convergence with the findings of Zhang et al. [20], which posited that strict glycemic control could improve pulmonary function. Both studies [19, 20] supported the notion that glycemic control is a significant modulator of lung health in individuals with diabetes. This internal focus on metabolic control as a key factor is similar to the findings of Klein et al. [18], who also considered intrinsic factors, albeit different ones. However, these studies [19, 20] differed from Khafaie et al. [16] and Kinney et al. [17], which centered on external risk factors. Zhang et al. [20] also observed that the duration of diabetes did not significantly affect pulmonary function, a finding that was not directly addressed by the other studies [16-19]. This aspect of the relationship between diabetes duration and lung function provides a novel perspective, suggesting that the reversibility of lung impairment may not be dependent on the longevity of the diabetic condition but rather on the management of glycemic levels.

Our research posited that individuals with undiagnosed or prediabetic conditions might have a heightened susceptibility to respiratory infections. This risk, we theorized, could manifest as increased episodes of COPD exacerbations or respiratory complications in those without COPD, particularly among individuals with a history of heavy smoking, whether current or past. Pulmonary surfactant protein D (SP-D), an integral component of the lung's innate immune defense, has been observed to be diminished in conditions such as obesity and type 2 diabetes [21]. Additionally, reductions in SP-D levels have been documented in smokers [22-24]. A correlation between decreased serum SP-D and COPD exacerbations has been proposed, potentially due to heightened lung permeability [22]. Research indicates that high blood sugar can weaken the body's primary defense mechanisms by reducing the effectiveness of beta-defensin-key peptides that protect against various pathogens in the lungs [23-24]. Kiselar et al. found that hyperglycemia increases the production of harmful compounds, compromising the antimicrobial activity of human β-defensin-2, which may heighten the risk of infections [24]. Studies have further shown that people with type 2 diabetes are more susceptible to infections leading to hospitalization, especially pneumonia [25-26]. Data from the Danish National Registry suggests that those with type 2 diabetes are more likely to require antibiotics, with an increased likelihood observed before and after diabetes diagnosis [26]. Cheng et al. [27] discuss how smoking and exposure to particulate matter can disrupt the gut microbiota and contribute to systemic inflammation and metabolic diseases, implicating the lung-gut axis in the progression of these disorders. They propose that targeting SCFA/GPCR signaling could help mitigate these effects, a novel approach not covered in our review. This is in line with the impact of environmental factors like air pollution on lung function in diabetics, as highlighted by Khafaie et al. and Kinney et al. [16, 17].

A meta-analysis by Díez-Manglano et al. found that type 2 diabetes is associated with declines in pulmonary function but not in the FEV1/FVC ratio. This contrasts with findings from Röhling et al., who reported an inverse correlation between HbA1c levels and the FEV1/FVC ratio, adding to the complex interplay between diabetes and lung function [28]. This complexity, including the noted heterogeneity in results not attributable to sex, BMI, smoking, or geography, supports the idea of multiple influencing factors, as also discussed by Klein et al. [18]. The findings from the review by Campagna et al. [29] contribute to an understanding of the intricate relationship between cigarette smoking, diabetes, and vascular complications. They emphasized the potential risk of developing incident diabetes among regular smokers and pondered the effects of smoking cessation on diabetes incidence and its progression. The review suggests that quitting smoking may not unequivocally reduce the incidence of diabetes or its complications, and they noted the potential negative impacts of quitting smoking, such as weight gain and poor glycemic control. These findings are somewhat at odds with typical expectations regarding smoking cessation, which are usually associated with improved health outcomes. The need for novel approaches to manage smoking cessation in diabetic patients was highlighted, signifying that traditional methods may not be fully effective or may have unintended consequences in this population. In comparison, Al-Ma'aitah et al. [30] conducted a systematic review and meta-analysis to examine patient-related factors influencing glycemic control in people with T2DM in Middle Eastern countries. Their findings supported the association between smoking and inadequate glycemic control, demonstrating an increased risk for smokers. This aligns with the general notion that smoking is a modifiable risk factor negatively impacting diabetes control and supports the findings of Campagna et al. [29] regarding the relationship between smoking and diabetes. Additionally, Al-Ma'aitah et al. [30] found that obesity, central adiposity, and longer disease duration were associated with poorer glycemic control, while physical activity and self-management practices were associated with better control. These findings are consistent with well-established diabetes management principles, which emphasize the importance of lifestyle modifications as part of diabetes care.

## Conclusions

The observations assimilated from this review point to the fact that DM significantly impacts pulmonary function, where individuals with diabetes experience a notable decline in lung function compared to those without the condition. This decline was influenced by a combination of intrinsic and extrinsic factors. Externally, environmental pollutants, specifically particulate matter, were significant contributors to respiratory impairment in the diabetic cohort. Smoking exacerbates pulmonary function decline, indicating that smokers with diabetes face a heightened risk of respiratory complications. The presence of obesity and advanced age further amplified this risk. Internally, metabolic control was a critical intrinsic factor affecting respiratory health. Poor glycemic control, indicated by high HbA1c levels, correlated with poorer pulmonary outcomes, especially in those with newly diagnosed diabetes. These results highlighted the importance of stringent glycemic management as a potential intervention to preserve lung function in individuals with diabetes. The review also revealed disparities in the impact of diabetes on pulmonary function across different demographics, with variations observed based on race and the coexistence of heart failure. This suggests that diabetes' respiratory effects are influenced by specific patient characteristics. The relationship between glycemic control and lung function emphasizes the need to maintain healthy blood glucose levels. Patients with type 2 diabetes, particularly those with recently diagnosed diabetes, should be informed of the need for glycemic control for respiratory health. Interventions targeted at improving HbA1c levels may benefit lung function and should be included in diabetes care plans. The discovery that diabetes duration does not have a substantial impact on pulmonary function suggests that treatments can begin at any stage of the disease. This underlines the possibility of improving lung function through stringent glycemic management, especially for people who have had diabetes for a long time. Therefore, it is recommended that clinicians encourage and support continuous glycemic management efforts in patients with diabetes, regardless of disease duration, in order to maximize pulmonary outcomes.

## Appendices

**Table 5:** Abbreviations used in the study

Abbreviation	Meaning
CRSs	Chronic respiratory symptoms
FVC	Forced vital capacity
FEV1	Forced expiratory volume in one second
FEV1%	Percent predicted forced expiratory volume in one second
FVC%	Percent predicted forced vital capacity
PM10	Particulate matter with a diameter of 10 micrometers or less
COPD	Chronic obstructive pulmonary disease
CI	Confidence interval
SD	Standard deviation
6MWD	Six-minute walk distance
PRISm	Patient-reported impact of sickness
BMI	Body mass index
DLCO	Diffusing capacity of the lungs for carbon monoxide
SNP	Single nucleotide polymorphism
VO2max	Maximum oxygen consumption
HbA1c	Hemoglobin A1c
hs-CRP	High-sensitivity C-reactive protein
